# Effectiveness and safety of enfortumab vedotin and pembrolizumab in a real-world patient population with urothelial carcinoma: results from a multi-institutional cohort (GUARDIANS)

**DOI:** 10.1007/s00262-026-04448-2

**Published:** 2026-07-12

**Authors:** Stefanie Zschäbitz, Irfan Bhatti, Jozefina Casuscelli, Ludwig Otto Pachmayr, Thomas Büttner, Thomas Elegeert, Nina Holzwarth, Severin Rodler, Martin Hennig, Emily Rinderknecht, Muammar Dib, Katrin Schlack, Christopher Darr, Anna-Lisa Volk, Philipp Schmucker, Friedemann Zengerling, Pia Paffenholz, Joschka Heil, Eva Erne, Subhajit Mandal, Frederik Wessels, Thea Maria Busse, Daniel Seidl, Susan Foller, Alexander Höllein, Tim Nestler, Christian Gratzke, Louis Rhode, Jakob Kohler, Günter Niegisch, Anna Katharina Seitz, Julia Heinzelbecker, Niklas Klümper, Margitta Retz, Viktor Grünwald, Can Aydogdu, Marco Julius Schnabel

**Affiliations:** 1 Department of Medical Oncology, National Center for Tumor Diseases (NCT), Heidelberg University Hospital, Im Neuenheimer Feld 460, 69120 Heidelberg, Germany; 2 Department of Urology, LMU University Hospital, LMU Munich, Munich, Germany; 3 School of Medicine and Health, Department of Urology, Technical University of Munich, TUM University Hospital, Munich, Germany; 4 Department of Urology, University Hospital Bonn (UKB), Bonn, Germany; 5 Department of Urology and Pediatric Urology, Saarland University Medical Center, Saarland University, Homburg, Saar Germany; 6 Department of Urology and Pediatric Urology, University Hospital Würzburg, Würzburg, Germany; 7 Department of Urology, University Hospital Schleswig Holstein, Kiel, Germany; 8 Hospital Am Urban, Berlin, Germany; 9 Department of Urology, Caritas St. Josef Medical Center, University of Regensburg, Regensburg, Germany; 10 Department of Urology, University Hospital Düsseldorf, Düsseldorf, Germany; 11 Department of Urology, University Hospital Muenster, Muenster, Germany; 12 Department of Urology, German Cancer Consortium (DKTK), University Hospital Essen, Essen, Germany; 13 Department of Urology, University Hospital Magdeburg, Magdeburg, Germany; 14 Department of Urology, University Hospital Freiburg, Freiburg, Germany; 15 Department of Urology Und Paediatric Urology, Hospital University of Ulm, Ulm, Germany; 16 Department of Urology, Uro-Oncology, Robot Assisted and Reconstructive Urologic Surgery, University of Cologne, Faculty of Medicine and University Hospital Cologne, Cologne, Germany; 17 Department of Hematology, Oncology, Palliative Care, Heilbronn Hospital, Heilbronn, Germany; 18 Department of Urology, Hospital University of Tübingen, Tübingen, Germany; 19 Department of Urology, University Hospital Marburg and Philipps University Marburg, Marburg, Germany; 20 Department of Urology and Urological Surgery, University Medical Centre Mannheim, Mannheim, Germany; 21 Department Hematology, Hemostaseology, Oncology and Stem Cell Transplantation, Medical University Hannover, Hannover, Germany; 22 Department of Urology, Diakonie-Klinikum Stuttgart, Stuttgart, Germany; 23 Urology Department, Jena University Hospital, Jena, Germany; 24 Department of Oncology, Red Cross Hospital, Munich, Germany; 25 Department of Urology, Bundeswehrkrankenhaus Koblenz, Koblenz, Germany; 26 Institute for Urologic Oncology, West-German Cancer Center Essen, University Hospital Essen and German Cancer Consortium (DKTK), Essen, Germany; 27 Present Address: Department of Urology, Heidelberg University Hospital, Heidelberg, Germany; 28 Present Address: Department of Urology, SRH Hospital, Gera, Germany; 29 Present Address: Veramed Hospital, Brannenburg, Germany; 30 Present Address: Department of Urology, University Hospital Marburg and Philipps-University Marburg, Marburg, Germany

**Keywords:** Bladder cancer, Enfortumab vedotin, Pembrolizumab, Real world evidence, Urothelial carcinoma

## Abstract

**Introduction:**

Enfortumab vedotin plus pembrolizumab (EVP) is the standard first-line treatment for metastatic or locally advanced urothelial carcinoma (m/laUC). Real-world patients frequently present with unfavorable clinical characteristics, raising uncertainty about whether outcomes mirror those of the pivotal EV-302/KEYNOTE-A39 trial. This study assessed effectiveness and safety of EVP in a real-world cohort of patients with m/laUC.

**Materials and methods:**

Retrospective multicenter study including patients treated with EVP across 25 German hospitals (between 03/2022 and 08/2025). Treatment delivery and monitoring followed local clinical standards. Adverse events (AEs) were documented per CTCAE v5.0. Responses were assessed locally using RECIST v1.1. Progression-free survival (PFS) and overall survival (OS) were analyzed using Kaplan–Meier methodology.

**Results:**

A total of 468 patients (median age = 69 years) were included. The overall response rate (ORR) was 50.2% (partial response:37.4%, complete response:12.8%). Median PFS was 10.2 months (95%CI,8.0–12.7). 12-month and 24-month OS rates were 71.9% and 60.6%, respectively. ECOG PS 0–1 and occurrence of skin toxicity were associated with improved outcomes. Any-grade AEs occurred in 81.8% and grade ≥ 3 AEs in 35.8%. Peripheral sensory neuropathy and skin toxicity were the most common AEs. Ten treatment-related fatal events were recorded.

**Conclusions:**

EVP demonstrated robust effectiveness and manageable toxicity in a large and diverse real-world cohort including patients with divergent histology. Skin toxicity was associated with superior ORR, PFS, and OS. Our findings support EVP as an effective first-line option for patients with m/laUC treated outside clinical trials.

**Supplementary Information:**

The online version contains supplementary material available at 10.1007/s00262-026-04448-2.

## Introduction

Urothelial carcinoma (UC), particularly in advanced or metastatic stages, remains one of the most challenging malignancies to treat, with long-term survival rates still poor despite continued advances in systemic therapies. Platinum-based chemotherapy has historically been the first-line standard of care (SOC); however, its efficacy is limited for many patients, especially those ineligible for cisplatin [1, 2]. Recent years have seen the emergence of novel agents such as enfortumab vedotin (EV), an antibody–drug conjugate (ADC) targeting nectin-4, and pembrolizumab, an immune checkpoint inhibitor (ICI) targeting PD-1, as promising new standards in the management of UC [3, 4].

The landmark phase III trial EV-302/KEYNOTE-A39, conducted by Powles et al., led to a paradigm shift: treatment with EV plus pembrolizumab (EVP) achieved significantly improved outcomes compared to platinum-based chemotherapy for previously untreated, metastatic or locally advanced UC (m/laUC) [5]. A total of 886 patients were randomized, with EVP yielding prolonged progression-free survival (PFS; median 12.5 vs. 6.3 months) and overall survival (OS; 31.5 vs. 16.1 months), with hazard ratios (HR) substantially superior to historical standards and tolerable toxicity profiles. Subsequent subgroup analyses demonstrated consistent benefit across all key patient subgroups, including both cisplatin-eligible and ineligible populations and regardless of baseline renal function or disease distribution [6]. Post-hoc analyses have further clarified the breadth of efficacy and safety, supporting wide applicability of this regimen regardless of patient age, performance status, specific sites of metastasis or nectin-4 status [7, 8].

Despite this robust body of evidence from controlled trials, real world data remain crucial to validate clinical trial observations and to assess the performance, safety, and tolerability of EVP in broader and less selected patient populations [9–11].

To our knowledge, the present analysis constitutes the largest retrospective, real world analysis reported to date, evaluating outcomes, adverse events (AE), and treatment patterns in first-line therapy for m/laUC across multiple academic practice settings. This study aims to complement and expand upon the EV-302 experience, offering additional insight into the use of EVP in everyday oncology practice.

## Materials (Patients) and methods

### Patient population and EVP management

Clinical data for patients with m/laUC treated with EVP in first-line were collected retrospectively from 25 community and university hospitals (in alphabetical order: Berlin, Bonn, Cologne, Düsseldorf, Freiburg, Essen, Hannover, Heidelberg, Heilbronn, Homburg/Saar, Jena, Kiel, Koblenz, Magdeburg, Mannheim, Marburg, Munich, Münster, Regensburg, Stuttgart, Tübingen, Ulm, and Würzburg; supplemental Table 1). Data was retrieved from patient charts by local investigators. Inclusion criteria included: patients with a diagnosis of locally advanced or metastatic urothelial carcinoma of the bladder, upper or lower urinary tract who received at least one application of EVP. Patients with tumors with divergent differentiation, subtype histology, or mixed histology were also eligible.

Imaging modalities, follow-up intervals, and therapy management followed the SOC at each institution. An EV dose of 1.25 mg/kg on days 1 and 8 in combination with pembrolizumab 200 mg qd21was considered the routine regimen. EV was approved by the European Medicines Agency (EMA) in August 2024 and thereafter reimbursed in Germany. Prior to this, patients received treatment based on individual reimbursement requests to the health insurance company. Responses were evaluated by local investigators according to Response Evaluation Criteria in Solid Tumors version 1.1. Treatment-related adverse events were recorded, and classified based on local investigator assessment at each participating center, using information available in the electronic health records. Toxicities were rated according to Common Terminology Criteria for Adverse Events (CTCAE) version 5.0 standards.

All procedures performed were in accordance with ethical standards of the institutional and/or national research committee and with the 1964 Declaration of Helsinki and its later amendments or comparable ethical standards. All patients consented to receive medical treatment according to local SOC. In cases of EVP treatment prior to EMA approval, separate informed consent to off-label drug use was provided. Any information connected to the identity of individual subjects was removed before study entry. The study was approved by the ethics committee of the University of Heidelberg (S-568/2022, last amendment 13.08.2024).

### Statistical analysis

Pseudonymized data were analyzed with RStudio and R version 4.5.1. Data processing was performed using the tidyverse framework, time-to-event analyses were conducted with the survival package, and tables and figures were produced with gtsummary, flextable, and ggplot2. Median follow-up was calculated using the reverse Kaplan–Meier method. Survival curves were estimated using the Kaplan–Meier method with step-function based 95% confidence intervals. Progression free survival (PFS) was defined as the time from EVP treatment initiation to disease progression or death from any cause, whichever occurred first. Overall survival (OS) was defined as time from EVP treatment initiation to death from any cause. Patients alive or without progression at the time of last follow-up were censored. Objective response rate (ORR) was defined as the proportion of patients achieving a complete response (CR) or partial response (PR) as best overall response, assessed by the treating investigator according to RECIST version 1.1. Disease control rate (DCR) was defined as the proportion of patients achieving CR, PR, stable disease (SD), or mixed response (MR) as best overall response. Skin toxicity was defined as any documented bullous or non-bullous exanthema during EVP treatment. Log-rank tests were used to compare survival distributions between groups. A two-sided *p *value of < 0.05 was considered statistically significant. Multivariable regression models were adjusted for the following covariates: age (< 75 vs. ≥ 75 years), ECOG performance status (0–1 vs. > 1), Bellmunt risk score (0–1 vs. ≥ 2), primary tumor site (bladder vs. upper tract urothelial carcinoma), histology (pure urothelial carcinoma vs. mixed/divergent/subtype), presence of liver metastases (absent vs. present), chronic kidney injury grade (0–1 vs. 2–3), platinum fitness (fit vs. unfit), presence of skin-related adverse events (absent vs. present), and disease extent (localized/ LN only vs. distant metastases).

## Results

### Baseline characteristics

**Fig. 1:**
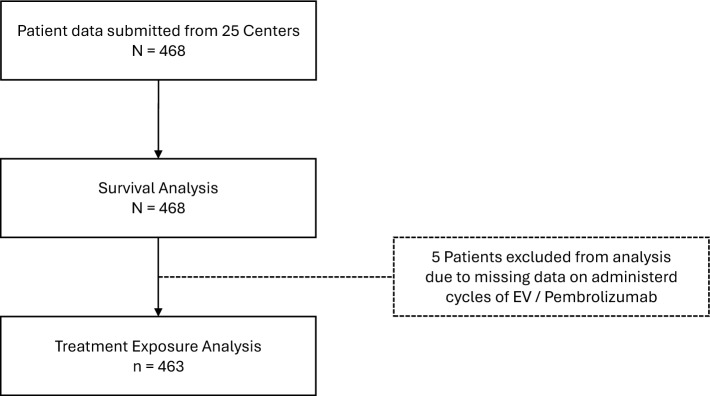
Consort diagram

We identified 468 patients (72.6% male) treated with EVP (Fig. 1; Table 1) who received at least one dose of EVP, with the first dose administered between March 2022 and August 2025. The median age at start of systemic treatment was 69 years (range: 25–90) and 32.1% of patients were aged 75 or above. ECOG performance scores were 0, 1, 2, 3, 4, or unknown in 47.6%, 32.1%, 16.4%, 2.8, and 0.2% patients, respectively. The bladder was the predominant primary tumor site (68.6%), followed by the upper urinary tract (25.2%). Primary histology was urothelial carcinoma in 91.9% of patients. Divergent differentiation or subtype histology was noted in 31 patients (n = 15 squamous cell carcinoma (SQC), n = 3 adenocarcinoma (AC), n = 2 small cell carcinoma (SCC), n = 11 other). At start of EVP, 53.3% of patients had more than one metastatic site. Lymph-node metastases were the most common localization (73.1%), followed by lung (36.57%), bone (28.0%), and liver (20.7%). According to the Bellmunt risk classification, 35.0% of patients had a risk score of 0, 4.6% had a score of 1, and 24.4% had scores ≥ 2. Data on previous and concurrent local therapies and previous (neo)adjuvant systemic therapies are displayed in supplemental Table 2; data on comorbidities in supplemental Table 3.

**Table 1:** Patient characteristics

Demographic characteristics	N = 468^1^
Age at start of EVP, years (range)	69 (25–90)
Age category (< 75 vs. ≥ 75 yr)	
< 75	318(67.9%)
≥ 75	150 (32.1%)
Sex	
Female	128 (27.4%)
Male	340 (72.6%)
Body mass index, kg/m^2^	25.1 (22.3–28.1)
Unknown	14
ECOG performance status	
0	223 (47.6%)
1	150 (32.1%)
2	77 (16.4%)
3	13 (2.8%)
4	1 (0.2%)
Unknown	4
Primary site of tumor	
Bladder	321 (68.6%)
Upper tract	118 (25.2%)
Urethra	4 (0.9%)
More than one primary site	24 (5.1%)
unknown	1 (0.2%)
Site of metastases	
Lymph node	342 (73.1%)
Lymph node only	132 (28.2%)
Lung	171 (36.5%)
Liver	97 (20.7%)
Bone	131 (28.0%)
Adrenal gland	18 (3.8%)
Brain	12 (2.6%)
Peritoneum	40 (8.5%)
Number of metastatic sites	
0	20 (4.3%)
1	199 (42.5%)
2	154 (32.9%)
≥ 3	95 (20.3%)
Primary histology	
Urothelial carcinoma	430 (91.9%)
Squamous cell carcinoma	16 (3.4%)
Adenocarcinoma	4 (0.9%)
Small cell carcinoma	2 (0.4%)
Other	14 (3.0%)
Unknown	2 (0.4%)
Bellmunt risk score (0–3)	
0	164 (35.0%)
1	190 (40.6%)
2	94 (20.1%)
3	20 (4.3%)
FGFR3 alteration	
No	65 (13.9%)
Yes	18 (3.8%)
unknown	385 (82.2%)

^1^Median (range); n (%)

EVP, enfortumab vedotin plus pembrolizumab

**Table 2:** Efficacy outcomes with enfortumab vedotin plus pembrolizumab

Endpoint	n/N	Result
Objective response rate (ORR)	235/468	50.2% (45.6–54.8%)
Complete response	60/468	12.8% (9.9–16.2%)
Partial response	175/468	37.4% (33.0–42.0%)
Stable disease	69/468	14.7% (11.7–18.3%)
Progressive disease	70/468	15.0% (11.8–18.5%)
No staging, due to clinical progression	29/468	6.2% (4.2–8.8%)
Staging scheduled, but not yet performed	65/468	13.9% (10.9–17.4%)
Disease control rate (DCR)	304/468	65.0% (60.4–69.3%)
Progression-free survival, median (95% CI), months		10.2 (8.0–12.7)
6-month PFS rate (95% CI)		64.6% (59.8–69.8%)
12-month PFS rate (95% CI)		45.5% (39.6–52.2%)
Overall survival, median (95% CI), months		33.7 (33.7–NR)
12-month OS rate (95% CI)		71.9% (66.6–77.6%)
24-month OS rate (95% CI)		60.6% (50.5–72.8%)

DCR, disease control rate; PFS, progression free survival; ORR, overall response rate; OS, overall survival

### Effectiveness outcomes

#### Progression free and overall survival

**Fig. 2:**
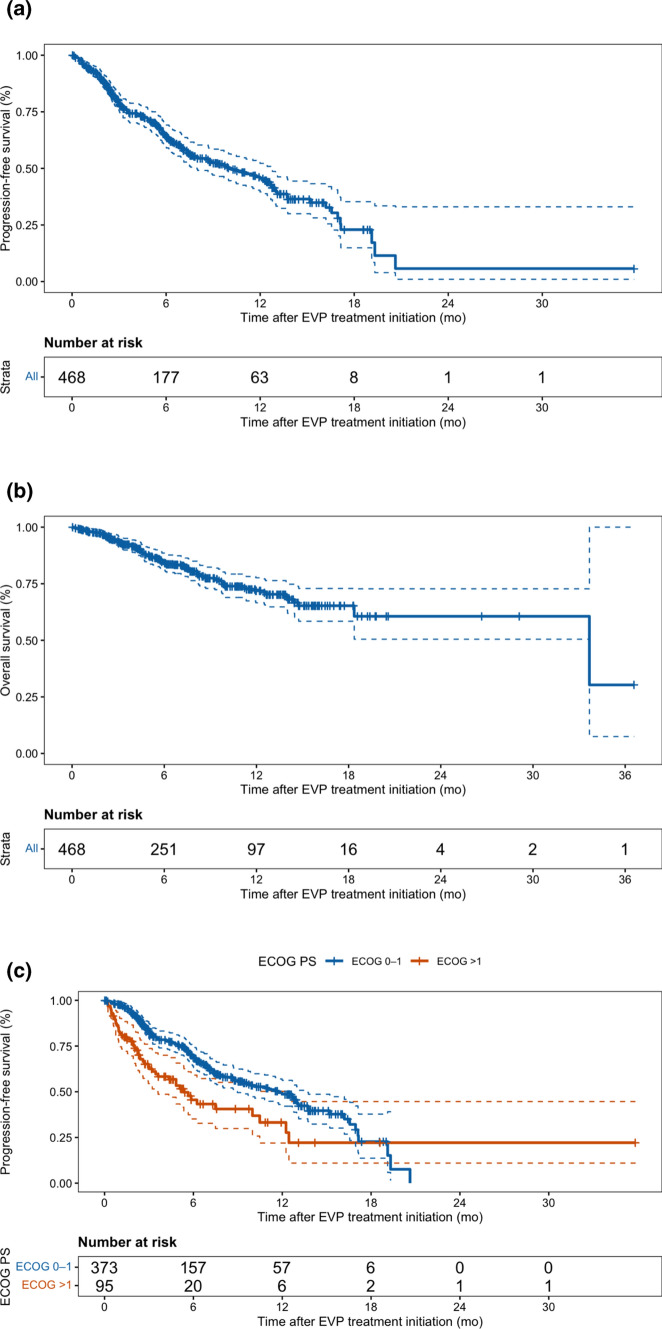

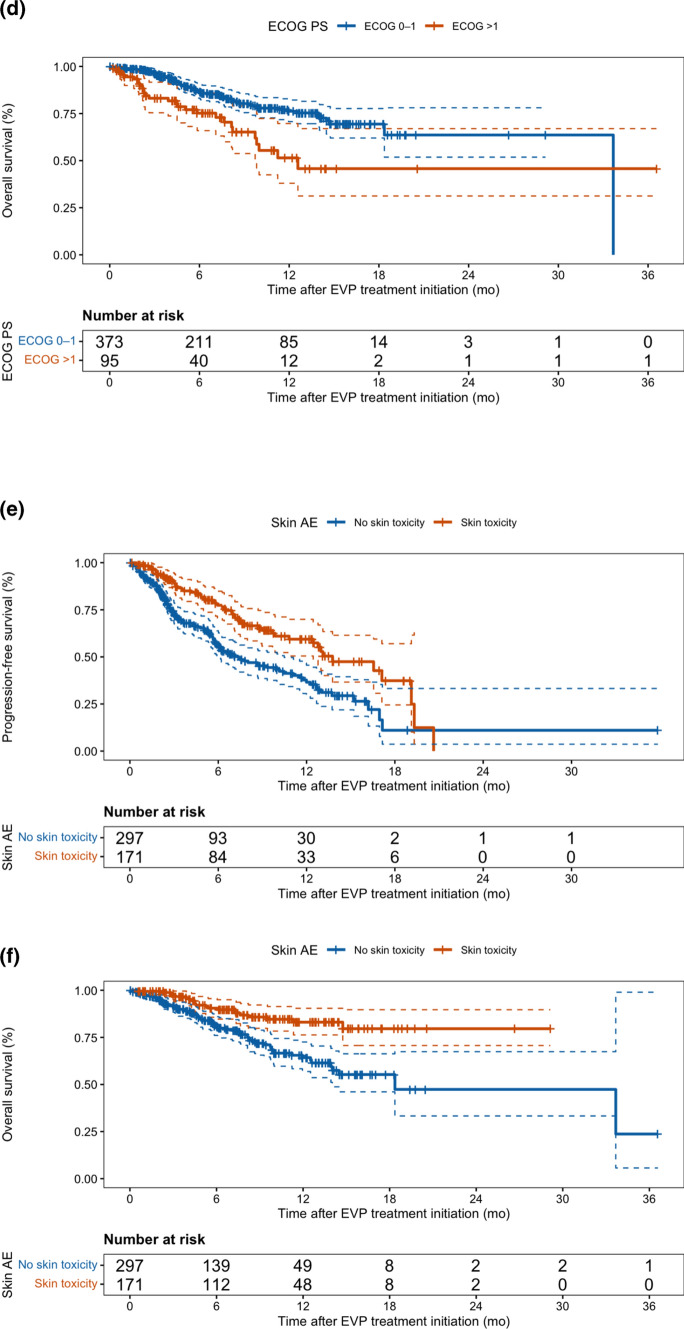

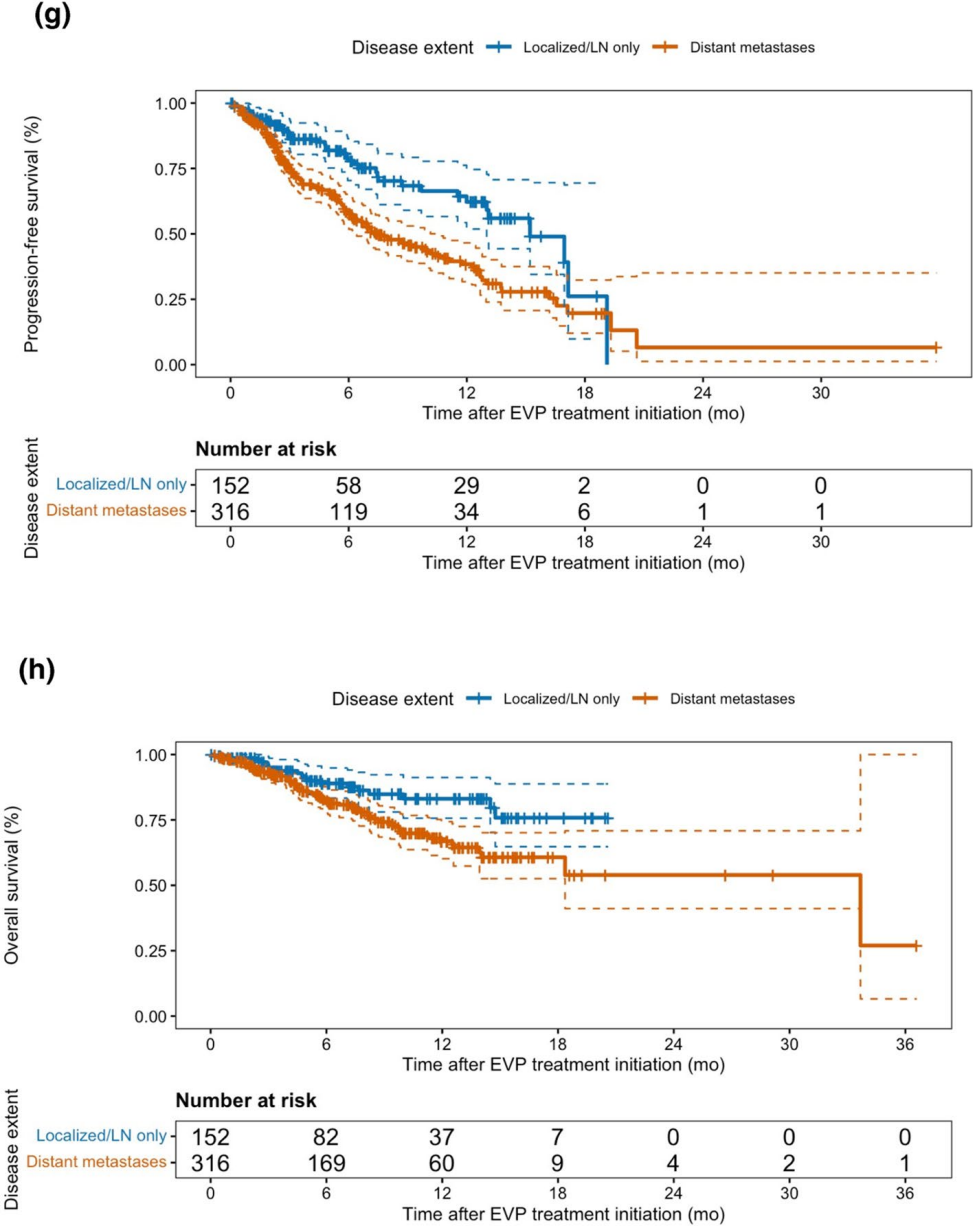
Survival analyses for patients treated with Enfortumab vedotin and pembrolizumab. **A** Progression free survival in the ITT population. **B** Overall survival in the ITT population. **C** Progression free survival and ECOG performance score. **D** Overall survival and ECOG performance score. **E** Progression free survival and skin toxicity. **F** Overall survival and skin toxicity. **G** Progression free survival in patients with locally advanced/lymph node only disease vs. extended disease. **H** Overall survival in patients with locally advanced/ lymph node only disease vs. extended disease

Median follow-up was 8.0 months (95% CI, 7.4–9.2; range: 0.0–36.6). Median PFS (mPFS) was 10.2 months (95% CI, 8.0–12.7; Fig. 2a). The 6-month and 12-month-PFS rates were 64.6% and 45.5%, respectively. When restricted to patients with at least one post-baseline staging assessment (n = 403) median PFS in this population was 11.0 months (95% CI: 8.0–13.0).

Median OS (mOS) was 33.7 months (95% CI, 33.7–not reached; Fig. 2b). The 12-month and 24-month-OS rates were 71.9% and 60.65% respectively.

Patients with ECOG 0–1 (n = 373) had a mPFS of 11.8 months (95% CI, 9.0–13.7) compared with 5.4 months (95% CI, 3.6–12.3) in patients with ECOG > 1 (n = 95; *p* < 0.001; Fig. 2c). mOS was 33.7 months (95% CI not reached) in patients with ECOG 0–1 versus 12.6 months (95% CI, 9.7–not reached) in patients with ECOG > 1 (< 0.001, Fig. 2d). Patients with skin toxicity (n = 171) demonstrated a mPFS of 13.8 months (95% CI, 12.5–not reached) compared with 7.4 months (95% CI, 6.0–10.5) in patients without skin toxicity (n = 297, *p* < 0.001, Fig. 2e). mOS was not reached in patients with skin toxicity versus 18.4 months (95% CI, 14.0–not reached) in patients without cutaneous toxicity (n = 297; *p* < 0.001, Fig. 2f). In patients with locally advanced and/or lymph node only disease (n = 152) mPFS and mOS were significantly longer compared to those patients with more extended disease (n = 316; mPFS: 15.2 months (95% CI, 13.0–NR) vs. 7.6 months (95% CI, 6.2–10.5); *p* < 0.001, Fig. 2g and mOS not reached vs. 33.7 months (95% CI, 18.4–NR); *p* < 0.01, Fig. 2h) in univariate analysis.

**Fig. 3:**
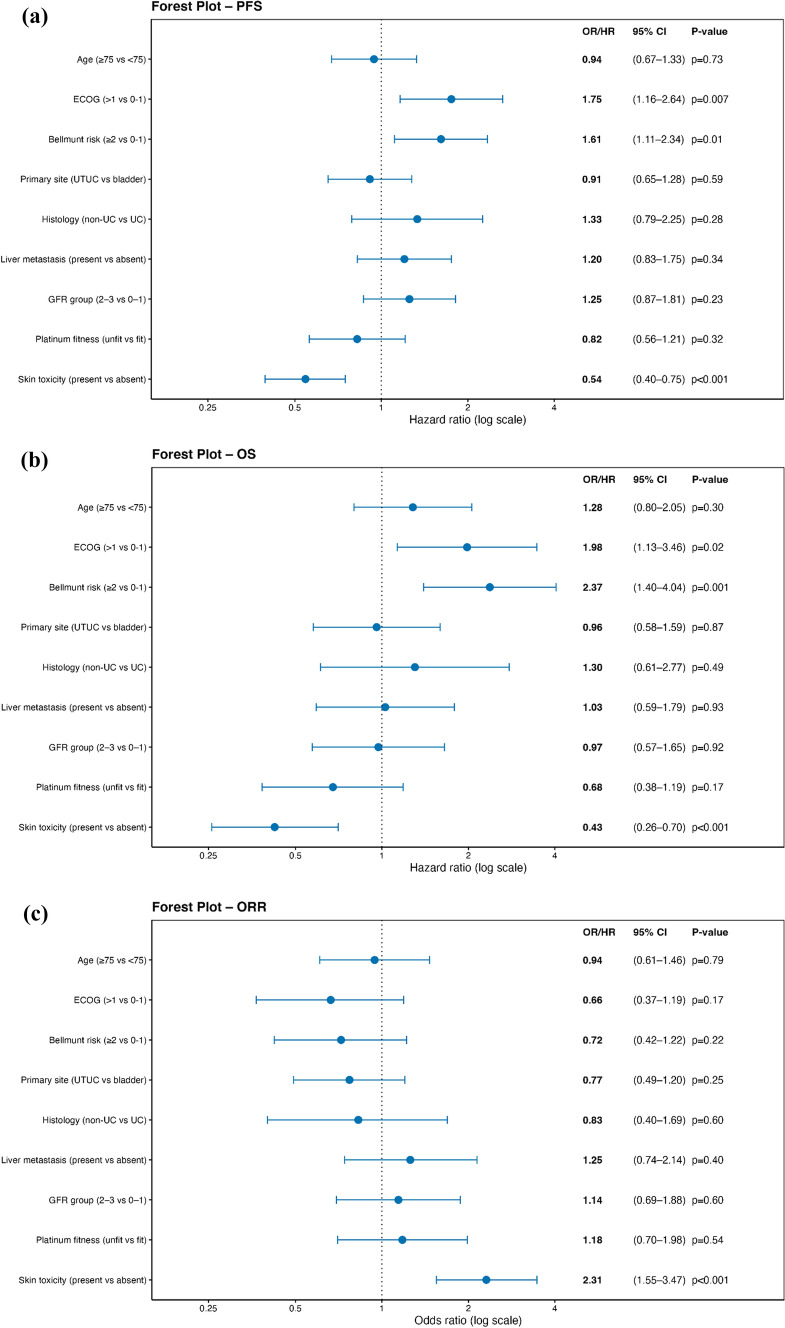
Forest plots of multivariate regression analyses for overall response rate, progression-free survival, and overall survival. Hazard ratios (HR) with 95% confidence intervals are shown. **A** Multivariable Cox proportional hazards regression analysis for progression-free survival (PFS). **B** Multivariable Cox proportional hazards regression analysis for overall survival (OS). **C** Multivariable logistic regression analysis for overall response rate (ORR)

Factors independently associated with worse PFS in the multivariable model included ECOG > 1 (HR 1.68, 95% CI, 1.12–2.53, *p* = 0.001), and Bellmunt risk score ≥ 2 (HR 1.60, 95% CI: 1.11–2.31, *p* = 0.01, Fig. 3a). Skin toxicity was independently associated with improved PFS (HR 0.56, 95% CI: 0.41–0.78, *p* < 0.001), same applied for disease extent “localized/lymph node (LN) only” (HR 0.57, 95% CI: 0.39–084, *p* = 0.004). Factors independently associated with worse OS included ECOG > 1 (HR 1.91, 95% CI: 1.09–3.34, *p* = 0.02), and Bellmunt risk score ≥ 2 (HR 2.27, 95% CI: 1.34–3.84, *p* = 0.002, Fig. 3b). Skin toxicity was independently associated with improved OS (HR 0.45, 95% CI: 0.27–0.74, *p* = 0.002).

#### Objective response

The objective response rate (ORR) was 50.2% (95% CI, 45.6–54.8), including 12.8% complete responses and 37.4% partial responses (Table 2). Stable disease as best response was observed in 14.7% of patients. Progressive disease occurred in 15.0% of patients, and in 6.2% of patients no further imaging was performed due to clinical deterioration. A subset of patients (13.9%) has not received staging imaging and was hence unevaluable for efficacy. The disease control rate (DCR) was 65.0% (95% CI, 60.4–69.3). The occurrence of skin toxicity was independently associated with higher ORR (OR 2.31, 95% CI: 1.53–3.43, *p* < 0.001. Figure 3c); the same was observed for extent of disease (OR 1.57, 95% CI: 1.01–2.44, *p* = 0.045). No other covariate in our multivariable regression model has reached statistical significance.

**Fig. 4:**
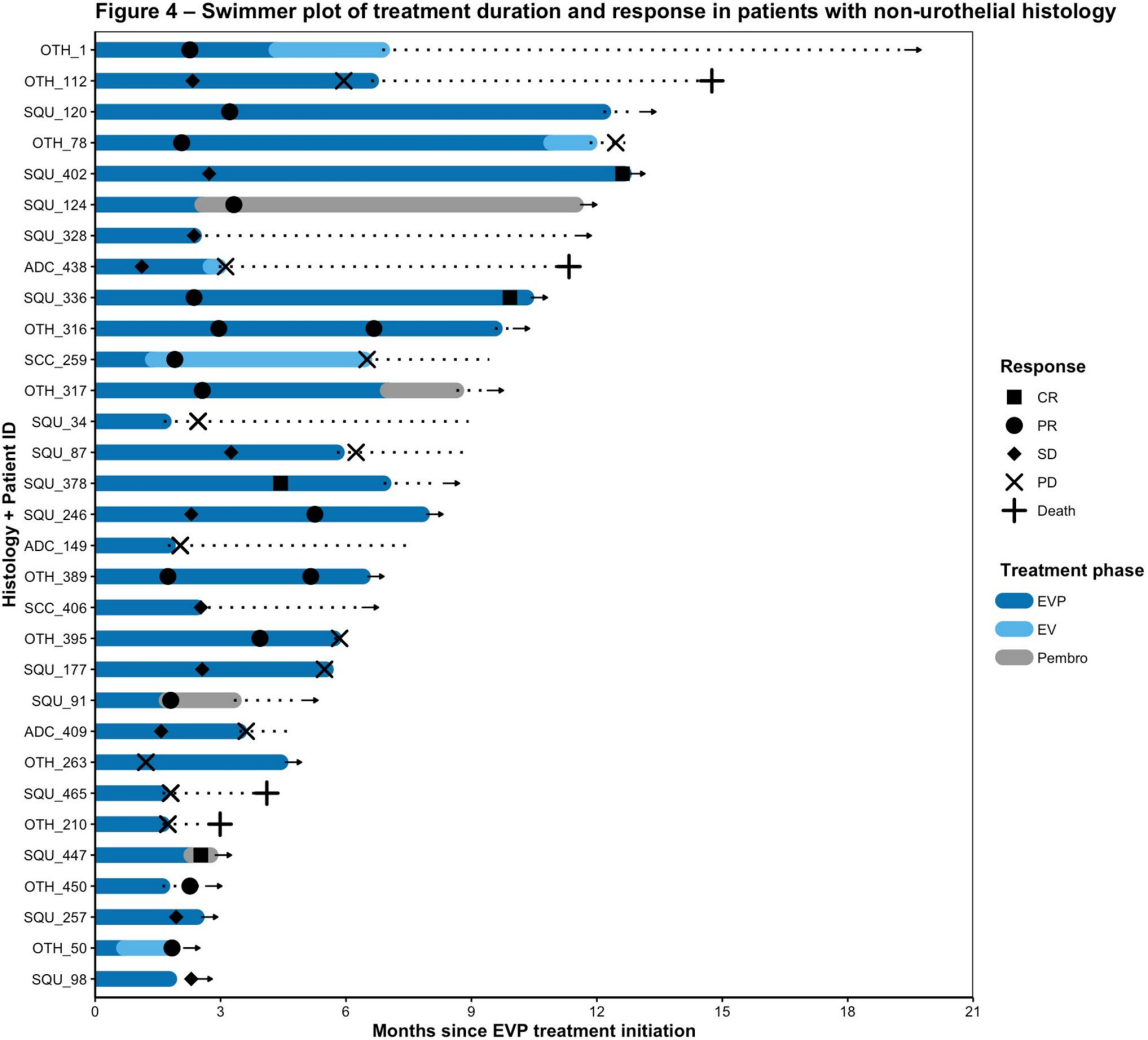
Swimmer plot of treatment duration and response in patients with tumors with divergent differentiation and subtype histologies. Each horizontal bar represents an individual patient (n = 31), sorted by follow-up duration from shortest (bottom) to longest (top). Patient identifiers include histology subtype prefix (SQU, squamous; ADC, adenocarcinoma; SCC, small cell carcinoma; OTH, other divergent differentiation and subtype/mixed). Colored bars indicate treatment phases: dark blue for combination therapy (EVP), light blue for enfortumab vedotin monotherapy (EV), and grey for pembrolizumab monotherapy. Dotted lines represent follow-up after treatment discontinuation. Black symbols mark tumor assessments: circle (partial response), square (complete response), diamond (stable disease), and X (progressive disease). Arrows ( →) indicate ongoing response at data cutoff. Plus symbols ( +) denote patient death. The plot demonstrates considerable heterogeneity in treatment duration (range: 1.6–19.3 months) and response patterns across non-urothelial histologies, with objective responses observed across all subtypes

In patients with tumors with divergent differentiation or subtype histology ORR was 51.6% (n = 16. CR: n = 4, 12.9% and PR: n = 12, 38.7%, Fig. 4), and DCR was 83.9% (n = 26). Response rates by histology were ORR 53.3%/DCR 86.7% for SQC; ORR 0%/DCR 66.7% for AC; ORR 50.0%/DCR 50.0% for small cell carcinoma, and ORR 63.6%/DCR 72.7% for “other”.

### Drug exposure and safety outcomes

Patients received a median of 9 administrations of EV (IQR 5–16) and 5 of pembrolizumab (IQR 3–10). The median duration of treatment (any component) was 5.5 months (IQR: 2.4–7.4, range: 1.6–12.6) and for the combination of EVP 3.2 months (IQR 1.7–6.5). Most patients (95.6% for EV and 95.2% for pembrolizumab) received the standard doses of drugs (i.e. 1.25 mg/kg of EV at days 1 and 8 and pembrolizumab 200 mg absolute on day 1 of a 21-days-cycle) at initiation. Disease progression remained the predominant reason for treatment discontinuation of both agents (26.6% for EV and 24.2% for pembrolizumab). Toxicity accounted for 17.1% and 13.7% of EV and pembrolizumab discontinuations, respectively. Dose reductions of EV occurred in 40.4% of patients. Pembrolizumab was interrupted by 31.5% of patients. Drug holidays were common, with 15.8%, 3.3%, and 22.5% of patients experiencing a pause in EV, pembrolizumab or both agents, respectively.

Treatment-related AEs were common but generally manageable. All-grade and grade 3–5 AEs were observed in of 383 patients (81.8%) and 168 patients (35.8%). All-grade and grade 3–5 immune related AEs were reported for 190 (40.6%) and 85 patients (18.2%), respectively. Systemic steroid use was applied in 123 patients (26.3%).

The most frequent AEs of any grade were sensory polyneuropathy (41.0%), exanthematous skin reactions without bullae (29.7%), pruritus (29.7%), fatigue (22.9%), anemia (20.3%), and weight loss (17.5%) (Supplemental Table 4). Gastrointestinal toxicities occurred in a subset of patients, including diarrhea (16.5%), nausea (8.8%), and constipation (7.3%). Most common immune- or inflammation-associated toxicities included hepatitis or elevated transaminases (14.5%), infections (14.5%), pneumonitis (6.2%), and hypothyroidism (5.3%).

Grade ≥ 3 AEs occurred in a minority of patients, with infections (6.6%), sensory polyneuropathy (5.6%), hepatitis/elevated transaminases (5.1%), and bullous or severe skin reactions (2.8%) being the most frequent high-grade events. Severe diarrhea, pneumonitis, and anemia were each observed in approximately 3% of patients.

In 10 patients treatment associated grade 5 toxicities were reported: infection (n = 3), gastrointestinal bleed (n = 2), cardiac toxicity (n = 2), pneumonia/pneumonitis (n = 1), and skin toxicity plus cardiac toxicity (n = 1).

### Subsequent therapy

Following discontinuation of EVP, 60 patients received at least one subsequent systemic therapy (Supplemental Table 5). Platinum-based chemotherapy remained the most common approach, with 5.1% receiving cisplatin plus gemcitabine and 3.0% carboplatin plus gemcitabine. Other agents included vinflunine (1.5%), FGFR inhibitors (1.5%), and taxanes (0.9%). Antibody–drug conjugates were used infrequently, with sacituzumab govitecan administered in 4 patients and trastuzumab deruxtecan in one patient.

## Discussion

In this large real-world cohort of 468 patients treated with EVP, we observed high and clinically meaningful antitumor activity together with a manageable safety profile. The observed ORR of 50.2%, including 12.8% CRs, aligns with outcomes reported in the pivotal EV-302/KEYNOTE-A39 trial and supports the clinical activity of EVP in a less selected population. mPFS was 10.1 months while mOS was 33.7 months; however the interpretation of OS is limited by the immaturity of the data. Notably, our cohort included patients with a broad spectrum of adverse baseline characteristics—including patients with ECOG > 2, advanced age, and a substantial proportion with multiple metastatic sites.

Recent real-world cohorts further support the activity and tolerability of EVP in routine clinical practice, although follow-up across studies remains limited. A Japanese multicenter cohort of 77 patients treated between September 2024 and September 2025 reported an ORR of 73.0%, with 6-month PFS and OS rates of 73.9% and 78.7%, respectively, after a median follow-up of 6.7 months [12]. Of note, 57.1% of the patients would have been ineligible for pivotal clinical trials, underscoring the importance of evaluating treatment outcomes in real-world patient populations. A large U.S. real-world cohort of 462 patients focused on outcomes according to baseline renal function and demonstrated that severe renal impairment (CrCl < 30 ml/min, n = 65 (14.1%)) was not independently associated with worse OS, PFS, or treatment interruption-free survival [13]. Similarly, a Mayo Clinic cohort including 183 patients reported median PFS values of 12.9 and 9.3 months for patients receiving EVP in first or subsequent treatment line, respectively [14]. In addition, an Austrian multicenter cohort of 203 first-line EVP-treated patients demonstrated an ORR of 63.6%, including CR in 21.5% of patients [15]. Median PFS and OS were not reached after a median follow-up of 5.8 months, again illustrating the immaturity of survival data in currently available real-world series. Inferior outcomes were associated with ECOG PS > 2 and higher comorbidity burden, consistent with our findings.

The safety profile observed in our cohort was broadly consistent with the known toxicities of EVP in clinical practice.While sensory neuropathy, cutaneous toxicity, pruritus, and fatigue were common, most AEs were grade 1 or 2 and consistent with the known toxicity profiles of both agents. High-grade hepatic events, infections, and severe skin reactions were the most notable serious toxicities; however, these remained within expected ranges. The frequency of treatment modifications—particularly EV dose reductions (40.4%) and pembrolizumab interruptions (31.5%)—highlights the importance of proactive toxicity management but did not appear to compromise overall outcomes, consistent with accumulating real-world data suggesting that dose adjustments do not diminish efficacy. Of note, the appearance of exanthema was associated with improved outcomes—both in ORR and survival. This observation aligns with findings from the EV302 cohort for EVP as well as real world populations for EV alone or in combination with pembrolizumab [16–18]. A key pharmacological feature of EV provides context for interpreting this association: EV delivers its cytotoxic payload via nectin-4, which is expressed not only on urothelial tumor cells but also on epidermal keratinocytes and sweat gland epithelium. This on-target/off-tumor activity produces skin reactions that typically early in the treatment course typically within the first one to two administrations. Hirotsu et al. reported a median time to rash onset of 27.8 days (range 7–52) in patients receiving EV, and a recent systematic review and meta-analysis that included data of 2554 patients treated with EV confirmed that EV-related skin toxicities “occur early in treatment” across clinical trials and real-world cohorts [19, 20]. These findings suggest that EV-associated skin toxicity is more likely a pharmacodynamic marker of near-immediate target engagement rather than a surrogate for prolonged treatment exposure. Nevertheless, as these observations are correlative, further investigation is needed to clarify their potential predictive significance.

Only 12.8% of patients received subsequent systemic therapy. While this may suggest a degree of disease control following EVP, the relatively short follow-up and the heterogeneity of the population—including a substantial proportion of de novo metastatic patients and those who had prior local therapy in the metastatic setting (11% surgery, 18% radiation)—limit definitive conclusions regarding durability. The relatively low use of later-line options such as sacituzumab govitecan, FGFR inhibitors, or taxane therapy may reflect patient selection, comorbidities, unresolved toxicities, or institutional treatment practices rather than long-lasting benefit alone. This observation aligns with prior real-world analyses indicating that intensive salvage therapy is often uncommon and less effective after modern combination stragies [21].

Several observations from our cohort further extend current knowledge. First, the high OS at 24 months (60.6%) in a population not restricted by clinical trial eligibility strongly supports the external validity of EVP across a broad and heterogeneous real-world population of patients with urothelial carcinoma, including divergent histologic subtypes. Second, the high proportion of older patients and those with upper-tract primary tumors reinforces EVP as a valuable regimen across clinically challenging subgroups. Third, the modest rates of immune-mediated events such as endocrinopathies, pneumonitis, or myocarditis reaffirm that combining an ADC with a ICI does not amplify immune toxicity. Finally, we observed that patients with locally advanced and/or lymph node–only disease experienced significantly prolonged mPFS and mOS compared with those presenting with more disseminated metastatic disease burden. This finding is biologically plausible and clinically highly relevant likely reflecting a lower systemic tumor burden and a disease biology more amenable to durable disease control. The substantially longer disease control observed in this subgroup may open a therapeutic window for consolidative or curative-intent approaches including definitive radiotherapy or radical surgery in a patient population currently deemed to be in palliative treatment situation. In this context, EVP may not only serve as a palliative first-line or neoadjuvant regimen, but also as an induction strategy in carefully selected patients. Jang et al. [22] reported on 6 patients with unresectable or oligometastatic UC that underwent radical cyctectomy in a curative intent after achieving CR to EVP. The CONSOLIdaTE-01 prospective single-arm single-center phase II trial will investigate 12-month PFS in approximately 41 patients with locally advanced unresectable (T4 and/or N+) or oligometastatic (</= 5 lesions) disease. Patients will receive 3 months of inductive EVP treatment followed by consolidative radical surgery of primary tumor and metastatic lesions. Defining the optimal patient selection, timing, and integration of local consolidative therapies in this setting represents an important area for future prospective research.

Our study has limitations inherent to real-world analyses, including short follow-up resulting in OS data immaturity, incomplete documentation for response assessments, heterogeneity in follow-up intervals, locally performed image review without central quality control, and potential underreporting of toxicities. Nevertheless, the large sample size and consistent treatment patterns across centers strengthen the robustness and representativeness of the findings. Importantly, the real-world mPFS and mOS data should be interpreted in the context of clinical practice, where imaging schedules and criteria for progression may differ from clinical trial standards.

In summary, our analysis presents the largest real-world evaluation of EVP to date and demonstrates high response rates, durable survival, and a manageable safety profile across a broad patient population. These findings complement and extend results from the pivotal trial, supporting EVP as the preferred first-line therapy for mUC and offering insights into tolerability, treatment modification patterns, and subsequent therapy utilization in clinical practice.

### Patient Summary

This study examined how well the combination of Enfortumab Vedotin and Pembrolizumab works for people with advanced bladder or urinary tract cancer in everyday clinical practice. Many patients showed good responses to the treatment, lived longer than expected, and most side effects were manageable. Interestingly, patients who developed skin reactions during therapy often experienced particularly strong benefits from the treatment.

## Supplementary Information

Below is the link to the electronic supplementary material.

Supplementary file1 (DOCX 17 KB)

## Data Availability

No datasets were generated or analysed during the current study.

## Abbreviations

ADC: Antibody drug conjugate; adeno carcinoma
AE: Adverse event
CR: Complete response
CTCAE: Common terminology criteria for adverse events
EMA: European medica agency
EV: Enfortumab vedotin
EVP: Enfortumab vedotin plus pembrolizumab
HR: Hazard ratio
ICI: Immune checkpoint inhibitor
m/laUC: Metastatic/locally advanced urothelial carcinoma
ORR: Overall response rate
OS: Overall survival
OTH: Other
PFS: Progression free survival
PD: Progression of disease
PNP: Peripheral polyneuropathy
PR: Partial response
SCC: Small cell carcinoma
SD: Stable disease
SOC: Standard of care
SQA: Squamous
UC: Urothelial carcinoma
