# Supplementary material for: Effectiveness and safety of enfortumab vedotin and pembrolizumab in a real-world patient population with urothelial carcinoma: results from a multi-institutional cohort (GUARDIANS)
Source: Cancer Immunol Immunother. 2026 Jul 12;75(7):184. doi: 10.1007/s00262-026-04448-2 (PMC13365084; doi:10.1007/s00262-026-04448-2)
Supplement: Supplementary file 1 — Supplementary file1 (DOCX 17 KB) [file 262_2026_4448_MOESM1_ESM.docx]

Supplemental table 1. Participating centers and patient recruitment

Supplemental table 2. Patient characteristics (local tumor therapies, neoadjuvant and adjuvant systemic therapies).

Supplemental table 3. Patient characteristics (comorbidities).

Supplemental table 4. Treatment-related adverse events (AE). Adverse events were classified as treatment-related based on local investigator assessment at each participating center, using information available in the electronic health records. Data reflect the frequency and severity of AEs observed in this multi-institutional retrospective cohort.

Supplemental table 5. Subsequent therapies.

Supplemental table 1.

| Center | Patients enrolled, n | First patient in | Last patient in | Patients enrolled, % of total |
| --- | --- | --- | --- | --- |
| Heidelberg | 58 | 2023-11-01 | 2025-08-14 | 12.4 |
| Bonn | 46 | 2023-12-18 | 2025-07-30 | 9.8 |
| München, LMU | 45 | 2023-11-28 | 2025-05-08 | 9.6 |
| München, TMU | 41 | 2024-01-11 | 2025-07-15 | 8.8 |
| Homburg | 32 | 2024-02-01 | 2025-03-18 | 6.8 |
| Kiel | 26 | 2024-02-05 | 2025-05-23 | 5.6 |
| Würzburg | 26 | 2024-01-09 | 2025-04-23 | 5.6 |
| Berlin | 25 | 2024-01-14 | 2025-06-18 | 5.3 |
| Regensburg | 25 | 2023-02-23 | 2025-04-03 | 5.3 |
| Düsseldorf | 22 | 2022-03-29 | 2025-04-11 | 4.7 |
| Münster | 22 | 2023-12-15 | 2025-01-20 | 4.7 |
| Essen | 18 | 2023-12-28 | 2025-05-08 | 3.8 |
| Freiburg | 15 | 2024-04-23 | 2025-04-25 | 3.2 |
| Magdeburg | 15 | 2023-04-03 | 2024-10-08 | 3.2 |
| Ulm | 9 | 2023-11-23 | 2024-09-05 | 1.9 |
| Köln | 8 | 2023-12-07 | 2024-09-02 | 1.7 |
| Heilbronn | 7 | 2023-04-04 | 2024-09-13 | 1.5 |
| Marburg | 6 | 2024-02-08 | 2024-09-12 | 1.3 |
| Tübingen | 6 | 2024-02-28 | 2025-04-08 | 1.3 |
| Mannheim | 5 | 2024-01-29 | 2024-06-05 | 1.1 |
| Hannover | 4 | 2024-06-04 | 2025-05-12 | 0.9 |
| Stuttgart | 3 | 2024-03-07 | 2024-04-11 | 0.6 |
| Jena | 2 | 2022-10-25 | 2023-12-27 | 0.4 |
| Koblenz | 1 | 2024-06-05 | 2024-06-05 | 0.2 |
| München, RKK | 1 | 2024-02-07 | 2024-02-07 | 0.2 |

Supplemental table 2.

| n (%) | **Overall** |
| --- | --- |
| **Prior treatment** | **N = 468** |
| **(Neo)adjuvant therapy** |  |
| None | 418 (89.3%) |
| Cisplatin/Gemcitabine | 48 (10.3%) |
| Cisplatin/Gemcitabine/Nivolumab | 1 (0.2%) |
| Other Chemotherapy + IO | 1 (0.2%) |
| **Adjuvant therapy** |  |
| None | 418 (89.3%) |
| Cisplatin/Gemcitabine | 28 (6.0%) |
| Cisplatin/Gemcitabine/Nivolumab | 1 (0.2%) |
| Other Chemotherapy + IO | 1 (0.2%) |
| IO mono | 13 (2.8%) |
| Carboplatin/Gemcitabine | 7 (1.5%) |
| **Local therapy** |  |
| none | 233 (49.6%) |
| Cystectomy | 161 (34.6%) |
| Nephroureterectomy | 50 (10.2%) |
| Radiotherapy | 5 (1.1%) |
| Chemoradiotherapy | 4 (0.8%) |
| Distant ureter resection | 6 (1.3%) |
| Cystectomy and nephroureterectomy | 4 (0.9%) |
| Partial cystectomy | 5 (1.1%) |
| **Surgery for metastases** | 52 (11%) |
| **Radiotherapy for metastases** | 83 (18%) |

Supplemental table 3.

| Comorbidities, n (%) | Overall N = 468 |
| --- | --- |
| Chronic kidney disease  Grade 5 | 272 (58.1%)  3 (0.6%) |
| Second malignancy | 104 (22.2%) |
| Diabetes | 88 (18.8%) |
| Arterial hypertension, vscular disease | 85 (18.2%) |
| Chronic heart failure | 76 (16.2%) |
| Arterial hypertension | 57 (12.2%) |
| Peripheral vascular disease | 54 (11.5%) |
| Lung disease | 50 (10.7%) |
| Status post myorcardial infarction | 48 (10.3%) |
| Preexisiting polyneuropathy | 33 (7.1%) |
| Status post stroke | 15 (3.2%) |
| Autoimmune disease | 15 (3.2%) |
| Eye disorder | 13 (2.8%) |
| Liver disease | 13 (2.8%) |
| Spinal ataxia, M. Parkinson | 3 (0.6%) |
| Status post kidney transplantation | 2 (0.4%) |

Supplemental table 4.

| AE | Any grade n (%) | Grade ≥3 n (%) |
| --- | --- | --- |
| Any | 383 (81.8%) | 168 (35.8%) |
| Any, immune related | 190 (40.6%) | 85 (18.2) |
| Sensoric polyneuropathy | 192 (41.0%) | 26 (5.6%) |
| Nonbullous exanthema | 139 (29.7%) | 17 (3.6%) |
| Pruritus | 139 (29.7%) | 2 (0.4%) |
| Fatigue | 107 (22.9%) | 11 (2.4%) |
| Anemia | 95 (20.3%) | 12 (2.6%) |
| Weight loss | 82 (17.5%) | 11 (2.4%) |
| Diarrhea | 77 (16.5%) | 14 (3.0%) |
| Hepatitis/ elevated transaminases | 68 (14.5%) | 24 (5.1%) |
| Infection | 68 (14.5%) | 31 (6.6%) |
| Alopecia | 56 (12.0%) | 2 (0.4%) |
| Hyperglycemia | 47 (10.0%) | 9 (1.9%) |
| Motoric polyneuropathy | 45 (9.6%) | 9 (1.9%) |
| Nausea | 41 (8.8%) | 0 (0.0%) |
| Constipation | 34 (7.3%) | 0 (0.0%) |
| Keratitis/ Dry eye | 34 (7.3%) | 1 (0.2%) |
| Bullous exanthema | 29 (6.2%) | 13 (2.8%) |
| Pneumonitis | 29 (6.2%) | 14 (3.0%) |
| Anorexia | 28 (6.0%) | 4 (0.9%) |
| Hypothyroidism | 25 (5.3%) | 1 (0.2%) |
| Emesis | 17 (3.6%) | 0 (0.0%) |
| Stomatitis | 17 (3.6%) | 2 (0.4%) |
| Arthritis | 16 (3.4%) | 1 (0.2%) |
| Dysuria | 15 (3.2%) | 0 (0.0%) |
| Leukopenia | 12 (2.6%) | 2 (0.4%) |
| Hypertension | 11 (2.4%) | 1 (0.2%) |
| Dizziness | 10 (2.1%) | 0 (0.0%) |
| Hyperthyroidism | 10 (2.1%) | 0 (0.0%) |
| Autoimmune nephritis | 9 (1.9%) | 4 (0.9%) |
| Hypercalcemia | 9 (1.9%) | 2 (0.4%) |
| Vertigo | 8 (1.7%) | 0 (0.0%) |
| Gastrointestinal bleeding | 6 (1.3%) | 3 (0.6%) |
| Thrombosis/Embolism | 6 (1.3%) | 1 (0.2%) |
| Autoimmune myocarditis | 5 (1.1%) | 5 (1.1%) |
| Hypophysitis | 5 (1.1%) | 4 (0.9%) |
| Gastric perforation | 3 (0.6%) | 3 (0.6%) |

Supplemental table 5.

| Subsequent therapy | (%) |
| --- | --- |
| Any | 60 (12.8) |
| Cisplatin + gemcitabine | 24 (5.1%) |
| Carboplatin + gemcitabine | 14 (3.0%) |
| Vinflunin | 7 (1.5%) |
| FGFR inhibitor | 7 (1.5%) |
| Taxane | 4 (0.9% |
| Sacituzumab govitecan | 3 (0.6%) |
| Cisplatin + etoposide | 2 (0.4%) |
| Carboplatin + paclitaxel | 1 (0.2%) |
| Carboplatin + paclitaxel + 5-FU | 1 (0.2%) |
| Cisplatin + gemcitabine + paclitaxel | 1 (0.2%) |
| Gemcitabine | 1 (0.2%) |
| Immune checkpoint inhibitor | 1 (0.2% |
| Sacituzumab govitecan + carboplatin | 1 (0.2%) |
| Trastuzumab deruxtecan | 1 (0.2%) |
| Other | 4 (0.9%) |
